# Editorial: Methodological, Theoretical and Applied Advances in Behavioral Spillover

**DOI:** 10.3389/fpsyg.2019.02701

**Published:** 2019-12-04

**Authors:** Christopher R. Jones, Lorraine Whitmarsh, Katarzyna Byrka, Stuart Capstick, Amanda R. Carrico, Matteo M. Galizzi, Daphne Kaklamanou, David Uzzell

**Affiliations:** ^1^Environmental Psychology Research Group, School of Psychology, University of Surrey, Guildford, United Kingdom; ^2^School of Psychology, Cardiff University, Cardiff, United Kingdom; ^3^Faculty of Psychology, Social Behavior Research Center, SWPS University of Social Sciences and Humanities, Wroclaw, Poland; ^4^Environmental Studies Program, University of Colorado at Boulder, Boulder, CO, United States; ^5^Department of Psychological and Behavioural Science, London School of Economics, London, United Kingdom; ^6^Department of Psychology, University of Portsmouth, Portsmouth, United Kingdom

**Keywords:** attitude, identity, behavior, spillover, theory, method, psychology, measurement

## Background

Psychology and allied disciplines (e.g., behavioral economics, marketing, and management) have established a range of techniques for understanding and changing behavior. Historically, the interventions derived from these techniques have largely focused on individual behaviors, rarely considering dynamic relationships between behaviors (i.e., whether the performance of a target behavior influences non-target^1^ behaviors). And yet work on response generalization (e.g., Ludwig, 2002), rebound effects (e.g., Greening et al., 2000), and moral licensing (e.g., Blanken et al., 2015) (to name but a few), has all variously described how changes in one behavior *can* have “knock-on” consequences for other actions.

Understanding secondary behavioral processes—including behavioral “spillover” effects—is a scientific and societal imperative. Scientifically, behavioral models and theories are improved by considering behavior beyond the narrow focus of a single action, offering a more comprehensive view of behavior change. Societally, interventions to address urgent problems, such as climate change or obesity, may be more effective and efficient if they are designed to change a suite of behaviors, rather than a single action.

The aim of this special issue is to unite contemporary psychological (and allied) research on the issue of behavioral spillover, to improve conceptual coherence in the field, and to advance knowledge in this area. In doing so, we hope to build upon the extant literature (for reviews see, Truelove et al., 2014; Dolan and Galizzi, 2015; Nash et al., 2017; Nilsson et al., 2017) to provide fresh insight into the underlying psychological mechanisms of the phenomenon, to explore cross-cultural similarities, and elucidate the principles underpinning effective intervention design.

This special issue comprises 14 conceptual, review, and empirical articles investigating a breadth of behavioral spillover research, both within behavioral domains and across socio-spatial, behavioral, and temporal contexts. The articles draw upon qualitative and quantitative methods to explore diverse theoretical and empirical aspects of spillover, including its measurement, the conditions under which it does and does not occur, and examples of where and when it can “backfire” (e.g., where “spillunder” might occur, Krpan et al.).

While this special issue does provide some conceptual depth and clarity to our understandings of spillover (e.g., its relationships with self-identity, Verfuerth et al.); and does advance the state-of-the-art regarding its measurement (Galizzi and Whitmarsh), it also raises many questions. A running theme in the studies is the unpredictable nature of spillover, unpredictability which serves to highlight important avenues for future research. As editors of this special issue we hope that the articles contained within may act as a source of “academic spillover;” informing the development of this field, such that the potential of spillover in responding to both scientific and social imperatives can be realized.

## Summary Of The Articles

This special issue comprises 14 articles, written by 40 authors spread across 11 countries and 4 continents. This section synthesizes and summarizes the focus and key findings of each article.

Three papers focus principally on the development of theory relating to spillover. Drawing on various psychological and economic theories (e.g., executive functioning, moral licensing and emotion regulation), Krpan et al. build a novel conceptual model of “spillunder” effects—where a person's intentions to act in accordance with a target intervention in the future (e.g., to exercise more) lead to performance of unintended actions in the present (e.g., overconsumption of food). Drawing upon attitude theory, Brügger and Höchli investigate the role that attitude strength plays in moderating the likelihood of spillover: requiring participants to think about past environmental or health behaviors before an opportunity to carry out successive goal-consistent actions, they find only limited evidence of spillover but some evidence for the anticipated moderation effect. Verfuerth et al. build and test a novel conceptual model of contextual spillover based upon Breakwell's Identity Process Theory (e.g., Jaspal and Breakwell, 2014): using the principles of identity integration, compartmentalization and conflict, they explore the mechanisms underpinning positive and negative contextual spillover, detail a real-world workplace intervention (centered upon dietary-choice), and reflect upon the theoretical and applied relevance of the findings derived from an affiliated qualitative interview-based study (incorporating a new visualization task).

Two papers focus on methodological contributions to the assessment of spillover. In a cross-cultural study comprising large samples from seven countries (Brazil, China, Denmark, India, Poland, South Africa, and the UK), Capstick et al. investigate individuals' beliefs about spillover processes, and assess the psychometric and cross-cultural properties of a new measure of behavioral spillover and its relation to subjective beliefs. Galizzi and Whitmarsh critically review experimental and non-experimental methods used to measure behavioral spillover and propose a systematic checklist designed to help researchers and policy-makers to rigorously and transparently test for behavioral spillover effects.

Several papers apply quantitative methods to understanding various forms of spillover. In order to learn more about the factors underpinning behavioral inconsistency in inter-context environmental action, Whitmarsh et al. use the Theory of Planned Behavior (Ajzen, 1991) as a framework for predicting waste-related behaviors at home, at work and on holiday: using a mixed-methods design, they reveal new insight into the strength and nature of inter-relationships between recycling and other waste-reduction behaviors within and between contexts. Fanghella et al. experimentally investigate the interaction between priming environmental self-identity and environmental action in the context of two commonly-used policy tools designed to change behavior (i.e., provision of social information and encouraging goal commitment): they urge caution when seeking to leverage environmental and other self-identities to promote behavior change and provide advice for those pursuing this strategy. Drawing upon principles of nudging (e.g., Thaler and Sunstein, 2003), Ghesla et al. note that research has yet to rigorously investigate whether nudges exert an impact upon non-target behaviors: focusing on pro-social behavior and the use of choice defaults, their experiment finds no evidence that negative spillover results from choice default nudging, and little evidence of positive spillover. In four lab experiments focused on environmental behaviors, Van der Werff and Steg explore the implications of pro-environmental vs. pro-economic messaging in yielding positive spillover: they find some evidence that environmental framing strengthens environmental identity and fosters spillover, while economic messaging weakens environmental identity and inhibits spillover.

Three papers employ longitudinal designs to assess the effects of behavior change campaigns. Thomas et al. use mixed methods to investigate behavioral and attitudinal responses to the introduction of a plastic bag charge in England: their results point to the broad, positive impact that the charge had on bag-use among the public, and evidence “policy spillover” in the form of enhanced support for policies to reduce plastic waste. Elf et al. identify the importance of social support for spillover and examine the emergence of spillover effects in response to an intervention led by a commercial partner, finding evidence of significant and sustained behavior and identity change and some evidence of spillover from an experimentally delivered intervention. Höchli et al. use an experimental field study to test the hypothesis that subordinate goals generated by short-term behavior change interventions are a potential source of negative spillover: examining a 2-month cycle-to-work campaign, they report upon some evidence of positive spillover and find no evidence of subordinate goals triggering negative spillover nor of their goal-level manipulation affecting the maintenance of post-intervention cycling behavior.

Finally, two papers apply qualitative approaches to provide a more in-depth exploration of the roots of behavioral spillover. Nash et al. explore subjective self-reflections of pro-environmental behavioral spillover in Brazil, China, and Denmark and discuss the prevalence and nature of within- and between-domain spillover effects via semi-structured interviews within a culturally-diverse sample: their findings not only point to commonalities in environmental spillover across countries, but also highlight the rarity of between-domain spillover and the link between pre-existing environmental values and the chance of “conscious” spillover occurring. Employing a series of life-history interviews with oil company workers, Uzzell and Räthzel, explore the processes by which practices are “carried over” between contexts: drawing on theories such as border crossing (Clark, 2000), they elucidate how myriad dispositional and situational influences govern and shape the transfer of environmental practices between places or contexts (in this case work and home).

## Author Contributions

All authors contributed to the writing of this editorial.

### Conflict of Interest

The authors declare that the research was conducted in the absence of any commercial or financial relationships that could be construed as a potential conflict of interest.

## Footnotes

^1^This could be the target behavior but in a non-targeted context, or non-targeted behaviors in the same or different contexts (e.g., Nilsson et al., 2017).
